# Association of *REL* polymorphisms and outcome of patients with septic shock

**DOI:** 10.1186/s13613-016-0130-z

**Published:** 2016-04-08

**Authors:** Julie Toubiana, Emilie Courtine, Frederic Tores, Pierre Asfar, Cédric Daubin, Christophe Rousseau, Fatah Ouaaz, Nathalie Marin, Alain Cariou, Jean-Daniel Chiche, Jean-Paul Mira

**Affiliations:** Medical School, Paris Descartes University, Paris, France; INSERM U1016, CNRS UMR 8104, Cochin Institute, Paris, France; Department of Pediatric and Infectious Diseases, Necker University Hospital, Assistance Publique-Hôpitaux de Paris, Paris, France; Bioinformatics Platform, Institut Imagine, Paris Descartes University- Sorbonne Paris Cité, 75015 Paris, France; Medical Intensive Care Unit, Angers University Hospital, Angers, France; Medical Intensive Care, Caen University Hospital, Caen, France; Intensive Care Unit, Cochin University Hospital, Assistance Publique-Hôpitaux de Paris, Paris, France

**Keywords:** cRel, *REL*, Genetics, Polymorphism, Septic shock, Multi-organ dysfunction syndrome, Mortality

## Abstract

**Background:**

cRel, a subunit of NF-κB, is implicated in the inflammatory response observed in autoimmune disease. Hence, knocked-out mice for cRel had a significantly higher mortality, providing new and important functions of cRel in the physiopathology of septic shock. Whether genetic variants in the human *REL* gene are associated with severity of septic shock is unknown.

**Methods:**

We genotyped a population of 1040 ICU patients with septic shock and 855 ICU controls for two known polymorphisms of *REL*; *REL rs842647* and *REL rs13031237*. Outcome of patients according to the presence of *REL* variant alleles was compared.

**Results:**

The distribution of *REL* variant alleles was not significantly different between patients and controls. Among the septic shock group, REL rs13031237*T minor allele was not associated with worse outcome. In contrast, REL rs842647*G minor allele was significantly associated with more multi-organ failure and early death [OR 1.4; 95 % CI (1.02–1.8)].

**Conclusion:**

In a large ICU population, we report a significant clinical association between a variation in the human *REL* gene and severity and mortality of septic shock, suggesting for the first time a new insight into the role of cRel in response to infection in humans.

## Background

Despite continued efforts and significant advances in critical care medicine, septic shock remains a significant health problem with a mortality rate around 35 % [1, 2]. Septic shock is defined as sepsis accompanied by cardiovascular failure that is often a part of multiple organ dysfunction syndrome (MODS) [3]. Thus, septic shock represents an extreme manifestation of the host inflammatory response to severe infection. Transcription of inflammatory mediators such as cytokines, chemokines, adhesion molecules and reactive oxygen species is strongly activated by the transcriptional factor NF-κB and contributes to the development of MODS [4, 5]. NF-κB is an ubiquitous family of inducible dimeric homodimer or heterodimer transcriptional factors composed by five members: Rel (c-Rel), RelA (p65), RelB, NF-κB1 (p50/p105) and NF-κB2 (p52/p100) [6].

The role of RelA in severe infection is well established, as it is highly recruited to the promoter of pro-inflammatory genes in non-survivors of septic shock [7, 8]. The cRel subunit was the least studied member of the Rel family, but seems to have also a critical role in the antimicrobial host defense. Indeed, in vivo studies revealed that cRel is required for macrophage activation [9, 10], adaptive immunity [11] and the control of lymphocyte proliferation [12, 13]. cRel is also a key regulator of numerous cytokines: IL-2, IL-3, IL-4, IL-6, IL-10, IL-13, IL-15, IL-21, IFN-γ, IFN-β, IFN-λ, MIP1-α and GM-CSF [9, 14–19]. More recently, in a murine model of polymicrobial sepsis, *Rel* deficiency led to an increased mortality, an enhanced systemic inflammatory response and a sustained depletion of spleen lymphoid dendritic cells [9–12, 20]. Furthermore, whole blood transcriptomics showed that cRel targets inflammatory and survival genes during sepsis [13, 20]. Moreover, genetic variants within the *REL* locus have been associated with inflammatory diseases or autoimmunity in Europeans [21]. Even if no study reports the importance of cRel in human sepsis, these elements highlight the potential importance of cRel for NF-κB targeted-immunomodulation in severe infections.

Recently, the role of genetic factors influencing the susceptibility to or the severity of severe sepsis has been extensively studied. Several single nucleotide polymorphisms (SNPs) have been characterized in genes of NF-κB pathway proteins. For instance, SNPs in *TLRs* [22–24], *TIRAP* [25], *IRAK1* [26, 27], *IκB* [28] and *NF*-*κB inducing kinase* (*NIK*) [29] genes have been associated with severity of sepsis. However, the association between genetic variants in *NF*-*κB* subunits and severe infections has been poorly reported. Hence, the present study aims to test the hypothesis of an association between clinically significant *REL* genetic variants and severity of septic shock in a large cohort of well-defined intensive care unit (ICU) patients.

## Methods

### Study population

This study was conducted prospectively in three medical ICUs in France. All three ICUs share similar severe sepsis management protocols based on international guidelines from the Surviving Sepsis Campaign for management of severe sepsis/septic shock [30]. The septic shock group was defined by usual criteria [31]. Briefly, patients were eligible for inclusion into the septic shock group (SS) if they had, within their stay in ICU, a clinical evidence of infection with two of four SIRS criteria (fever (>38.2 °C) or hypothermia (<36 °C); tachycardia (>90 beats/min); tachypnea (>20 breaths/min) or need of mechanical ventilation; white cell count >12 × 10^9^/L) and if, after an adequate fluid resuscitation, they required vasopressor infusion (norepinephrine, epinephrine or dopamine >8 μg/kg/min) to maintain a mean arterial pressure higher than 60 mmHg. Exclusion criteria included comorbidities highly associated with death in SS [32]: age above 85 years, cardiac failure (NYHA class III or IV), liver insufficiency (child C), bone marrow aplasia or leucopenia not related to septic shock (white blood cell count <0.50 × 10^9^/L), immunosuppression (HIV, current immunosuppressive therapy including steroids with equivalent prednisone >0.5 mg/kg per day) or ongoing cancer with undergoing treatment.

The control group (C) was composed of patients hospitalized simultaneously in the three ICUs for other reasons than infection and who did not develop sepsis nor required any inotropic or vasopressor agents during their ICU stay. Similar exclusion criteria were used for the control and the SS groups.

Patients were followed up throughout their ICU stay, and clinical and biological characteristics were prospectively collected: age, gender, SAPSII score and previous medical history of severe infection requiring hospitalization. For the SS group, characteristics of current infectious episode were also collected: primary sites of infection, infection-related microorganisms, development of multi-organ dysfunction syndrome (MODS) (defined as the presence of more than two organ system failures occurring simultaneously ICU stay) [3], mechanical ventilation requirement estimated by ventilator-free day (VFD: time without mechanical ventilation within the ICU period censured to 28 days) [33] and ICU mortality. To minimize confounding factors due to ethnical differences, all patients selected in the study were Caucasians and had European origins.

### Ethics approval

The Institutional Review Board of Cochin Hospital, Paris, France, approved the study, and informed consents have been obtained from the patients or their relatives.

### Determination of genotypes, DNA isolation and allelic discrimination

Two previously described SNP have been analyzed. The SNP rs842647 is a A → G transition located in the second intron of *REL* gene on chromosome 2 (chromosomic location 61119471). The SNP rs13031237 is a G → T transition located in the fourth intron of *REL* gene (chromosomic location 61136129). All genetic analyses were performed blinded from the clinical data. Genomic DNA was extracted from mononuclear cells using MagNA Pure Compact automate (Roche Diagnostics®). DNA extracts were then quantified and stored in code-barr tubes (2DCYPHER, ABgene®) to maintain anonymous status of the patients all along the study. Real-time PCR allelic discrimination assays were realized by TaqMan® method on Abi 7900 (Applied Biosystems®). Probe and primer combinations were designed to discriminate the two *REL* SNPs (rs842647 and rs13031237). Quality control for genotyping was performed by automatic sequencing 12 patients carrying the different *REL* genotypes in order to confirm allelic discrimination results and also by re-genotyping 20 % of the entire cohort. All DNA samples showing discrepancy between the two analyses were definitively sequenced (*n* = 4).

### Statistical analysis

All data were analyzed by SPSS v11.5 and “R” v3 softwares. Both SNPs were tested for Hardy–Weinberg disequilibrium to check for stratification. In order to calculate the Šidák multiple testing correction, we first evaluate the effective number of independent tests (called Meff) in the analysis by using the methodology proposed by Li and Ji [34]. This method aims to prevent from overcorrection due to possible linkage disequilibrium (LD) between the SNPs.

Power calculation has been based on the frequency of the variant allele in the control population as proposed by Hattersley et al. [35]. Hence, for an incidence of the variant allele of 11 % in the control population and a power at 90 %, a 50 % increase in the case population with a type I error of 5 %, 886 individuals in each group appear to be sufficient to detect genetic susceptibility to SS (http://www.stat.ubc.ca/~rollin/stats/ssize/b2.html). For the second study assessing the prognostic value of the variant alleles in SS group, given the frequency of the variant genotype, and an expecting mortality rate at 35–40 % in the SS subgroup group, we considered that 870 SS patients were sufficient for a power at 0.90 (type I error at 0.05) to identify a 30 % difference in genotype frequency.

Descriptive results of continuous variables were expressed as median and interquartile range reflecting population distribution. Variables were tested with Chi-square test for categorical data (sex, multi-organ failure, primary sites of infection, microorganisms, genotypes) and with Mann–Whitney *U* test for numerical data (age, SAPSII, VFD).

A multivariate logistic regression model was used to determine the respective role of *REL* genotypes for susceptibility to SS and to ICU mortality. Confounding factors with a *p* value <0.05 were included in this model. Continuous variables were included without any transformation, and genotypes were considered as a factor (dichotomous unordered variate) to avoid the implicit dose effect when coding the genotypes 0, 1 and 2 according to the number of mutated alleles carried. Results were expressed as odds ratio (OR) and 95 % confidence interval (CI), and variable with *p* value <0.05 was defined as statistically significant.

## Results

### Patient characteristics

The total enrolled Caucasian population was composed by 1040 septic shock patients (SS) and 855 controls (C). In the C group, enrolled ICU patients were admitted for various non-infectious reasons (metabolic: 39 %, neurological: 29 %, respiratory: 22 %, cardiovascular: 6 % and surgical: 4 %) and did not develop severe sepsis and did not require vasopressor infusion during their ICU stay. C patients were younger than SS patients (46 vs. 66 years, respectively, *p* = 0.001), and females were more represented in C group (45 and 39 %, for C and SS, respectively, *p* = 0.006). Mortality rate and occurrence of MODS in the C group were 3 and 1 %, respectively.

All SS patients received norepinephrine or epinephrine as first vasopressor. The main site of infection was the lung (58 %); microorganisms were identified in 74 % of the cases, mainly Gram-positive bacteria. Median SAPSII value of 54 and high percentage of patients with multiple organ dysfunctions (61 %) underlined the severity of the septic shock population. The ICU mortality rate of the SS group was 30 %.

### *REL* genotypes and septic shock susceptibility

Hardy–Weinberg proportions were comparable to expected percentages regarding *REL* variants: *REL rs842647* (*p* = 0.9) and *REL rs13031237* (*p* = 0.7) in favor of homogeneity of population ethnicity. To determine whether *REL rs842647* and *rs13031237* SNPs were associated with septic shock susceptibility, genotype frequencies were determined for SS and C patients. As reported in Table 1, no significant difference was found between the two groups. Moreover, these incidences were similar to those reported in the HapMap database-reported genotype distribution for European population (http://www.ncbi.nhm.nih.gov/projects/SNP/snp_viewTable.cgi?pop=1409).

**Table 1:** *REL* genotype frequencies

Genotype (%)	Minor allele^a^	Heterozygous	Major allele^a^	*p* value^b^
*REL rs842647*				
Control (*n* = 855)	8.9	42.2	48.9	
Septic shock (*n* = 1040)	9.7	42.9	47.4	0.74
*REL rs13031237*				
Control (*n* = 855)	13.5	44	42.6	
Septic shock (*n* = 1040)	11.4	46	42.6	0.38

^a^Homozygous genotype

^b^ *p* value control versus septic shock group for each genotype

### *REL* genotypes and outcome of septic shock

Among the SS patients, general clinical characteristics were not significantly different between patients carrying *REL* minor allele and patients homozygous for major allele on both *REL*-analyzed loci (Table 2). In order to study the link between *REL* SNPs and septic shock severity, we compared acute respiratory distress syndrome (ARDS) and MODS frequencies, and VFD value between patients carrying REL rs842647*G and rs13031237*T minor alleles and in those homozygous for the major alleles. As given in Table 2, VFD values were not different between these different groups of patients. The presence of rs13031237*T allele was not associated with higher risk of ARDS or MODS (Table 2). However, MODS occurred more frequently in patients carrying the REL rs842647*G allele [*p* = 0.04, OR 1.3, 95 % CI (1–1.7), Table 2]. For multiple testing correction, we calculated a Meff of 1.75 [34] leading to a corrected *p* value of 0.06. A similar trend was observed for ARDS (*p* = 0.08, Table 2).

**Table 2:** Clinical characteristics of SS group by *REL* genotype

Characteristics	*REL rs842647*		*REL rs13031237*	
	G/G or A/G	A/A^a^	T/T or A/T	T/T^a^
	*n* = 493	*n* = 547	*n* = 443	*n* = 597
Age^c^	65 [52; 77]	67 [54; 77]	66 [53; 76]	65 [52; 77]
Men (%)	60.5	62.3	61.1	61.6
SAPSII^c^	55 [43; 67]	53 [42; 67]	54 [43; 68]	54 [41; 66]
Diabetes (%)	22.1	17.3	19.9	19.7
COPD (%)	13.2	17.4	15.7	14.4
Septicemia (%)	27.7	29.9	29.5	27.7
Gram-positive bacteria (%)	49.9	47.2	48.1	49.2
VFD^c^	8 [0; 20]	11 [0; 21]	10 [0; 20]	10 [0; 20]
ARDS (%)	38.6	33.5	34.8	37.9
Multiple organ dysfunctions (%)	63.9	57.8^b^	60.8	61.3

^a^Homozygous for major allele

^b^Patients carrying minor allele and homozygous for major allele were compared: *p* value <0.05

^c^Continuous variables were expressed as median and interquartiles [25th percentile; 75th percentile]

### *REL* genotypes and mortality of septic shock

**Fig. 1:**
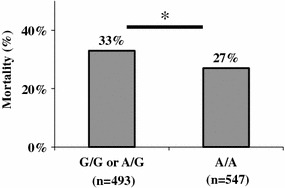
rs842647*G allele is associated with increased mortality. G/G or A/G represented patients with septic shock who carried the variant rs842647*G allele. A/A represented patients with septic shock who were homozygous for the rs842647*A major allele. *p = 0.03

In SS group, mortality was not significantly different between patients carrying REL rs13031237*T minor allele and homozygous for the major allele of this SNP (30.2 vs. 29.8 %, for minor and major alleles, respectively, *p* = 0.9). In contrast, the presence of the rs842647*G minor allele was significantly associated with a higher mortality rate (33 vs. 27 %, for minor and major alleles, respectively, *p* = 0.03) (Fig. 1). Multivariate logistic regression analysis confirmed the importance of the rs842647*G minor allele for mortality risk during septic shock [OR 1.4, CI (1.02–1.8), nominal *p* value = 0.03]. For multiple testing correction, the calculated Meff of 1.75 [34] led to a corrected *p* value of 0.05, which reaches near significance.

## Discussion

The present study showed that septic shock patients carrying the rs842647*G minor allele had an over risk of MODS and mortality. In contrast, no association was found between the REL rs13031237*T allele and the severity of septic shock.

This study was the first to investigate the importance of two polymorphisms within *REL* gene in a large European population of septic shock patients. Several human studies have suggested that these variants may have an effect on the inflammatory balance, as they have been associated with inflammatory and autoimmune diseases. Indeed, the intronic *rs13031237* SNP in the *REL* gene was associated with susceptibility to rheumatoid arthritis [36–38] and psoriasis [39]. The intronic *rs842647* SNP was linked to a higher risk of Crohn’s disease, ulcerative colitis [40] and celiac disease [41, 42] and primary sclerosing cholangitis [43]. Genome-wide studies have also found that *REL* locus was associated with psoriasis [37, 44], rheumatoid arthritis [37], ulcerative colitis [45, 46] and Hodgkin’s lymphoma [47]. However, functional and structural effects of these polymorphisms are still unknown and need to be investigated. Given that variant alleles are located on an intronic site, it is possible that these polymorphisms affect transcriptional efficiency of *REL* gene or these variants may be in strong linkage disequilibrium with a variant inside a neighbor gene.

The higher rate of mortality observed in SS patients carrying rs842647*G might be linked to a higher inflammatory state, as they also developed more frequently MODS. They also tend to have more ARDS and lower VFDs however not significant, but this is most likely underpowered, as VFDs are not normally distributed. Mortality in septic shock was partially related to hyper-activation of NF-κB [7, 48]. In this setting, previous genetic studies on several gain of function SNPs in genes of receptors and signaling molecules upstream of NF-κB, such as TLR1 and IRAK1 [22, 26], showed a significant association with severity of sepsis. These genetic factors might unbalance the fine-tune regulation toward a hyper-inflammatory deleterious state. However, the exact role of cRel on inflammatory processes is less understood in humans. Recent studies have shown that cRel could be involved in autoimmunity, such as inflammatory arthritis [49] and autoimmune encephalomyelitis [50]. More recently, cRel was shown to have a key role in antimicrobial defense processes. *Rel*−*/*− mice are more susceptible to *Leishmania major* [9] or *Toxoplasma gondii* infections [10], to viral infection by *Influenza virus* [11], to bacterial infection by *Listeria monocytogenes* [12] and to polymicrobial sepsis [20]. cRel is probably important in pro-/anti-inflammatory balance as *Rel*−*/*− mice seemed to have an enhanced inflammatory response [20].

The study design quality is important for a right interpretation of genetic association studies [35]. We tried to follow closely these quality criteria. First, it is important to select a SNP of a protein involved in the physiopathology of the disease. As already mentioned, *REL* seems to be an interesting gene to study because NF-κB plays a central role in physiopathology of sepsis, and recent studies show the importance of cRel in this context. However, one important limit of our study is the absence of data regarding the functional effect of these two SNPs. Functional data are needed to improve our understanding of how rs842647*G variant of REL is related to sepsis severity. Second, the population homogeneity has been controlled by limiting the study on European patients without severe comorbidities. The third item is probably one of the more controversial in the sepsis field: choice of a clearly defined phenotype to avoid confusion factors. Thus, we have selected only patients with septic shock whose diagnosis and treatment are standardized [3], and these patients had no major comorbidity or immunosuppressive treatment, severe autoimmune diseases in particular, that could have been confounding the results. However, it is impossible to rule out effects of confounding factors or gene–environment interactions in our results, and septic shock is heterogeneous with regard to the source of infection. Sample size is essential for statistics quality in association study but is difficult to achieve in pure septic shock population. At our knowledge, our cohort is one of the largest ever published populations in this topic and is large enough to diminish type I error. It is important to consider that after correction for multiple testing, our result only reached near significance. Šidák/Bonferroni correction assumes, however, that markers are independent, whereas the SNPs studied here are in LD and are therefore not truly independent from each other. As a result, though we tried to take into account LD by the calculation of Meff, the adjustment is likely to overcorrect in this case. We therefore consider that our preliminary results would need a validation in independent cohorts. Finally, this genetic association study is limited to one gene. Genome-wide association studies (GWAS) are now discovering new unsuspected genes that might have an impact on sepsis outcome [51].

## Conclusion

The association between rs842647*G allele and severity of septic shock brings a new perspective on the role of cRel subunit of NF-κB in severe infections in humans. Better understanding of the genetic effects of NF-κB-dependent inflammatory pathways is essential for further research on modulation of NF-κB activity by specific inhibitors, such as small molecule inhibitors of cRel, as an adjuvant treatment for sepsis [52, 53]. Further studies are needed to investigate the functional role of this *REL* polymorphism on the inflammatory processes observed in sepsis and to validate these encouraging results in independent cohort.

## Abbreviations

MODS: multiple organ dysfunction syndrome
IL: interleukin
IFN: interferon
SNP: single nucleotide polymorphisms
ICU: intensive care unit
SS: septic shock group
C: control group
VFD: ventilator-free day
ARDS: acute respiratory distress syndrome
LD: linkage disequilibrium
TLR: Toll-like receptor
